# Evaluation of integrated interventions layered on mass drug administration for urogenital schistosomiasis elimination: a cluster-randomised trial

**DOI:** 10.1016/S2214-109X(19)30189-5

**Published:** 2019-06-26

**Authors:** Stefanie Knopp, Bobbie Person, Shaali M Ame, Said M Ali, Jan Hattendorf, Saleh Juma, Juma Muhsin, Iddi S Khamis, Khalfan A Mohammed, Jürg Utzinger, Elizabeth Hollenberg, Fatma Kabole, David Rollinson

**Affiliations:** a Swiss Tropical and Public Health Institute, Basel, Switzerland; b University of Basel, Basel, Switzerland; c Wolfson Wellcome Biomedical Laboratories, Department of Life Sciences, Natural History Museum, London, UK; d Schistosomiasis Consortium for Operational Research and Evaluation, University of Georgia, Athens, GA, USA; e Public Health Laboratory-Ivo de Carneri, Pemba, Tanzania; f Neglected Diseases Programme, Ministry of Health, Zanzibar, Tanzania; g Schistosomiasis Control Initiative, Department of Infectious Disease Epidemiology, Faculty of Medicine, Imperial College London, London, UK

## Abstract

**Background:**

Elimination of schistosomiasis as a public health problem and interruption of transmission in selected areas are targets set by WHO for 2025. Our aim was to assess biannual mass drug administration (MDA) applied alone or with complementary snail control or behaviour change interventions for the reduction of *Schistosoma haematobium* prevalence and infection intensity in children from Zanzibar and to compare the effect between the clusters.

**Methods:**

In a 5-year repeated cross-sectional cluster-randomised trial, 90 shehias (small administrative regions; clusters) in Zanzibar eligible owing to available natural open freshwater bodies and public primary schools were randomly allocated (ratio 1:1:1) to receive one of three interventions: biannual MDA with praziquantel alone (arm 1) or in combination with snail control (arm 2), or behaviour change activities (arm 3). Neither participants nor field or laboratory personnel were blinded to the intervention arms. From 2012 to 2017, annually, a single urine sample was collected from approximately 100 children aged 9–12 years in the main public primary school of each shehia. The primary outcome was *S haematobium* infection prevalence and intensity in 9–12-year-old children after 5 years of follow-up. This study is completed and was registered with the ISRCTN, number 48837681.

**Findings:**

The trial was done from Nov 1, 2011, through to Dec 31, 2017 and recruitment took place from Nov 2, 2011, until May 17, 2017. At baseline we enrolled 8278 participants, of whom 2899 (35%) were randomly allocated to arm 1, 2741 (33%) to arm 2, and 2638 (32%) to arm 3. 120 (4·2%) of 2853 in arm 1, 209 (7·8%) of 2688 in arm 2, and 167 (6·4%) of 2613 in arm 3 had *S haematobium* infections at baseline. Heavy infections (≥50 eggs per 10 mL of urine) were found in 126 (1·6%) of 8073 children at baseline. At the 5-year endline survey, 46 (1·4%) of 3184 in arm 1, 56 (1·7%) of 3217 (odds ratio [OR] 1·2 [95% CI 0·6–2·7] *vs* arm 1) in arm 2, and 58 (1·9%) of 3080 (1·3 [0·6–2·9]) in arm 3 had *S haematobium* infections. Heavy infections were detected in 33 (0·3%) of 9462 children.

**Interpretation:**

Biannual MDA substantially reduced the *S haematobium* prevalence and infection intensity but was insufficient to interrupt transmission. Although snail control or behaviour change activities did not significantly boost the effect of MDA in our study, they might enhance interruption of transmission when tailored to focal endemicity and applied for a longer period. It is now necessary to focus on reducing prevalence in remaining hotspot areas and to introduce new methods of surveillance and public health response so that the important gains can be maintained and advanced.

**Funding:**

University of Georgia Research Foundation Inc and Bill & Melinda Gates Foundation.

## Introduction

Schistosomiasis is a parasitic disease caused by infection with blood flukes of the genus *Schistosoma*.^1^ An estimated 800 million people are at risk of infection and more than 200 million people are infected.^2^ In 2016, the global burden of schistosomiasis was 1·86 million disability-adjusted life years.^3^ Over the past 15 years, substantial progress has been made in the control of schistosomiasis. There has been a shift from morbidity control towards elimination in selected areas and new targets have been issued by WHO: elimination of schistosomiasis as a public health problem (prevalence of heavy intensity infections below 1% in all sentinel sites) and interruption of transmission (reduction of incidence of infection to zero) in selected areas by 2025.^1^, ^4^ The Zanzibar archipelago, offshore from Tanzania, is one of the first settings in sub-Saharan Africa targeted for elimination of urogenital schistosomiasis as a public health problem and interruption of transmission.

The cornerstone of schistosomiasis control is mass drug administration (MDA) with praziquantel, but moving towards elimination will require complementary measures.^4^, ^5^, ^6^ Suggested measures to reach interruption of transmission in selected areas where transmission is low and highly focal include intensified treatment (ie, treatment intervals shorter than 12 months and targeting not only school-aged but also preschool-aged children and adults, including women of reproductive age) plus intermediate host snail control, improvements in access to clean water, sanitation, and hygiene (WASH) to reduce and ideally impede the contamination of freshwater bodies, and interventions to assist people in changing their behaviour to prevent transmission and infection.^4^, ^7^, ^8^, ^9^, ^10^

**Research in context**

**Evidence before this study**

Elimination of schistosomiasis has been shown to be feasible. In 2011, the 56th World Health Assembly called on all countries endemic for schistosomiasis to intensify control interventions and to strengthen surveillance, with the aim of eliminating the disease. In 2012, WHO set elimination of schistosomiasis as a public health problem and interruption of transmission in selected areas as targets for 2025. Countries having achieved interruption of transmission reported economic improvements, the integrated use of mass drug administration (MDA), intermediate host snail control, or improved access to clean water, sanitation, and hygiene. A large-scale concurrent research trial of strategies to control *Schistosoma mansoni* done in St Lucia from 1965 to 1981 showed best results when chemotherapy was supplemented by snail control or new household level water supplies. Meta-analyses highlight that control of intermediate host snails can contribute significantly to moving towards schistosomiasis elimination in high-risk areas. However, evidence for strategic decisions based on results from randomised trials is absent.

**Added value of this study**

We did a 5-year cluster-randomised trial to assess the effect of different interventions for elimination of urogenital schistosomiasis as a public health problem and interruption of transmission. Biannual MDA with praziquantel was offered to all age groups with the exception of children below the age of 3 years across the Zanzibar islands. New behavioural interventions were developed in a human centred design approach and applied in randomised communities. The capacity for snail control was established. In randomised communities, water bodies containing intermediate host snails were targeted by focal mollusciciding. Our trial showed that biannual MDA applied alone or in combination with snail control or behaviour change activities can substantially reduce the overall *Schistosoma haematobium* prevalence and infection intensity. Urogenital schistosomiasis was eliminated as a public health problem from Zanzibar in more than 90% of the shehias included in the study, but transmission is not yet interrupted and reinfection occurs. Although randomised additional interventions in our study did not significantly boost the effect of MDA, they might enhance interruption of transmission when tailored to focal endemicity and applied for a longer period.

**Implications of all the available evidence**

Schistosomiasis is a focal disease. In settings where elimination as a public health problem and interruption of transmission is the goal, intervention strategies need to be tailored to the local micro-epidemiology and culture. It is now necessary to build on the experience gained in this trial and other studies, to focus on reducing prevalence and intensity in remaining hotspot areas, and to introduce new methods of rigorous surveillance, followed by specific public health response so that the important gains can be maintained and advanced.

Our objectives were to assess biannual MDA applied alone or with complementary snail control or behaviour change interventions for the reduction of *Schistosoma haematobium* prevalence and infection intensity in children from Zanzibar and to compare the effect between the clusters.

## Methods

### Study design and participants

The Zanzibar archipelago consists of two main islands: Pemba and Unguja. Each island is divided into districts, which are subdivided into small administrative units called shehias. In 2012, the national census recorded 121 shehias in Pemba and 210 shehias in Unguja. The total population is estimated at 1·3 million. Urogenital schistosomiasis caused by infection with *S haematobium* has been highly prevalent in the past century, with prevalences exceeding 50% in some places, but was reduced to an overall prevalence below 10% in 2012.^11^, ^12^, ^13^, ^14^ It is hence important to note that our trial was done in a setting that had been exposed to MDA with praziquantel for several years and that our baseline population in 2012 was mostly not naive to treatment.^13^

The study was a 5-year cluster-randomised open-label trial with three intervention arms. The study design has been published elsewhere.^15^ We included children aged 9–12 years. From 2012 to 2017, annually, a single urine sample was collected from approximately 100 children aged 9–12 years in the main public primary school of each of the 90 study shehias. A shehia was defined as the cluster and intervention unit. The trial was done in 90 shehias on Pemba and Unguja, from Nov 1, 2011, through to Dec 31, 2017, and recruitment took place from Nov 2, 2011, until May 17, 2017. Interventions in all arms started within one year after the baseline survey in 2012 and were intensified until the endline survey in early 2017. The first community-wide treatment MDA round was conducted on April 28, 2012. Snail control started on Aug 1, 2012. Behaviour change interventions started in a phase-in approach from Nov 1, 2012.

Ethical approval was obtained from the Zanzibar Medical Research Ethics Committee in Stonetown, Zanzibar (ZAMREC; reference no. ZAMREC 0003/Sept/011), the Ethikkommission beider Basel (EKBB) in Basel, Switzerland (reference no. 236/11) and the Institutional Review Board of the University of Georgia in Athens, GA, USA (project no. 2012-10138-0). Written informed consent was obtained from the parents or guardians of participating children.

### Randomisation and masking

Stratified by island, shehias were randomly allocated to one of three intervention arms (ratio 1:1:1), as described elsewhere.^15^ In brief, 15 shehias on each island received biannual MDA with praziquantel administered by the Neglected Tropical Diseases (NTD) Programme of the Zanzibar Ministry of Health across the archipelago (arm 1); 15 shehias received snail control in addition to biannual MDA (arm 2); and 15 shehias received behaviour change interventions in addition to biannual MDA (arm 3). Owing to the nature of the intervention, neither participants nor field or laboratory personnel were blinded to the intervention arms.

### Outcomes

The primary outcome was *S haematobium* infection prevalence and intensity in 9–12-year-old children in Zanzibar in 2017 after 5 years of follow-up at individual and cluster level. The primary outcome was reworded after registration of the study to meet the appropriate definition of a variable^16^ and to point out the main trial population. The change in the primary outcome was based on the recommendation of trialists who supported the preparation of the statistical analysis plan (appendix) and decided upon by the trial leadership and the Schistosomiasis Consortium for Operational Research and Evaluation (SCORE) secretariat. The decision to reword the primary outcome was done before the statistician had access to the data for analysis. Secondary outcomes including the *S haematobium* prevalence and intensity in first-year students and adults are presented elsewhere.^17^ No outcomes were excluded from the analyses.

### Procedures

The baseline survey was done in the primary schools of the 90 study shehias in early 2012, with annual follow-up surveys done in early 2013, 2014, 2015, 2016, and 2017. The purpose and procedures of the study were explained to eligible children. Once we received the informed consent form signed by the parents or guardians, the participants were provided with a plastic container and instructions for urine collection between 09:00 h and 14:00 h the following day. A single urine sample from each participant was transferred to the laboratory of the NTD Programme in Unguja or to the Public Health Laboratory—Ivo de Carneri in Pemba. Each urine sample of sufficient quantity was visually examined for macrohaematuria, for microhaematuria by means of reagent strips (Haemastix; Siemens Healthcare Diagnostics GmbH, Camberley, Surrey, UK), and for *S haematobium* eggs, by means of the filtration method.^17^ 10% of all urine samples were re-read by a senior laboratory technician for quality control. In the months following the survey, MDA was done in schools and communities and praziquantel (40 mg/kg) was offered to the whole eligible population. Treatment coverage data were collected as described elsewhere in detail.^18^

Praziquantel was administered biannually across both islands, in all shehias located in Pemba and Unguja, with the exception of the South district and the Urban A and B subdistricts in Unguja.^17^ In community-wide treatment (CWT), implemented twice per year from April, 2012 onward, praziquantel was distributed by trained community drug distributors (CDDs) to the whole eligible population, excluding children younger than 3 years, children treated during school-based treatment (SBT), severely sick people, and pregnant women.^18^ In additional SBT, implemented for the first time in MDA round 4, praziquantel was administered to schoolchildren by teachers by means of a dose pole and the intake of drugs was directly observed. Data on treatment coverage of CWT was collected from the records of CDDs and of SBT from teachers, by staff of the Zanzibar Ministry of Health. Our project staff collected additional data on treatment coverage and compliance during the annual cross-sectional surveys in schools and communities.^16^, ^18^

For snail control activities, human water contact sites (HWCSs) were identified in the 30 study shehias before and over the course of the trial with the help of local knowledge and information. Trained teams did surveys for intermediate host snails (*Bulinus* spp) at each HWCS multiple times per year outside of the heavy rainy season. For this purpose, approximately 15 m of the shoreline were measured and searched for snails of all species by two collectors for 15 min, using their hands and snail scoops.^19^ The molluscicide niclosamide (Bayluscide; donated by Bayer SAS, Monheim, Germany) was sprayed at HWCSs only if *Bulinus* spp were present.^17^ Niclosamide wettable powder was mixed with pond water (according to manufacturer's instructions) and applied to the shoreline around the HWCS with Hudson backpack sprayers or a petrol power spraying machine, depending on the environment. The HWCS's location, type, water chemistry, presence of snails, and niclosamide spraying were recorded at each survey.

Community co-designed behaviour change interventions were developed and implemented in the 30 study shehias in a staggered approach by trained teams.^20^ Classroom-based and school-wide intervention components were done by trained primary school teachers and religious school teachers using culturally tailored, interactive tools, materials, and engagement methods developed within the programme (eg, flipcharts, blood fluke pictures, snail boards, and self-drawing of schistosome life-cycles) to teach children about schistosomiasis transmission and prevention.^20^, ^21^ Teachers and children did regularly, school-wide, Kichocho Day Events incorporating dramas, poems, and games that focused on schistosomiasis transmission, prevention, and treatment. Parents and other community members were encouraged to participate in Kichocho Day Events and interactive health education activities. Community-based interventions included community meetings, evening educational films, and the construction of one male and one female urinal per shehia near a freshwater body with known schistosomiasis transmission. In the second half of the project, community co-designed washing platforms were constructed in close proximity to a safe water source in behavioural shehias with the highest disease prevalence. Data on school census and children exposed to the interventions as well as community intervention components were collected over the course of the implementation process.

### Statistical analysis

The sample size calculation, eligibility criteria, and randomisation procedures of clusters and study participants are described in the published study protocol.^15^ In brief, to reach a desired power of 80%, the sample size of clusters (ie, shehias) exceeded the total number of schistosomiasis-endemic shehias in Unguja and Pemba and the sample size of participants was logistically not feasible. Hence, the choice of 15 shehias per intervention arm per island, and the number of people to be tested was a compromise between what was considered optimal and what was practically achievable. Participants were considered *S haematobium*-positive if the urine filtration method revealed at least one *S haematobium* egg per 10 mL urine, or, in the absence of a urine filtration result, if microhaematuria was detected with reagent strips. Infection intensities were classified into light (1–49 eggs per 10 mL urine) and heavy (≥50 eggs per 10 mL urine) according to WHO thresholds.^22^ Egg counts were truncated at 1000 eggs per 10 mL urine.

The absolute and relative difference (% change) in the *S haematobium* prevalence at baseline in 2012 and endline in 2017 were calculated. Arithmetic mean (AM) egg counts, including zeros, were calculated at baseline and endline as a proxy for transmission force at shehia level; AM egg counts, excluding zeros, were calculated at baseline and endline as a proxy for transmission force at individual level. The AM egg reduction rate from 2012 to 2017 was calculated by means of the following formula: 1–AM egg counts in 2017/AM egg counts in 2012. Generalised estimating equation models with binary logit functions and negative binomial distributed outcomes with log link functions, and independent correlation structure were applied to compare trial arms. All models used robust variance estimators to account for correlation within clusters (ie, the school). Biannual MDA alone was the designated reference group. For unadjusted estimates, only infection status (as outcome) and treatment arm were included in the model. In the adjusted analysis, sex and age were included in the model as explanatory variables. In addition, the observations in the adjusted analysis were weighted by the inverse cluster size (probability weights), which ensures that each cluster contributes equally to the generalised estimating equation, regardless of its size. Intra-class correlation was established by means of mixed models consistent with the generalised estimating equation, setup in the primary analysis.

Given the relatively high number of clusters, balance in baseline characteristics was a reasonable assumption. Since we detected some discrepancy in baseline prevalence among the three trial arms, we complemented the results with an exploratory analysis using different types of adjustment for baseline prevalence. Treatment coverage was calculated as described elsewhere in detail.^18^

Descriptive statistics were done by means of Stata IC 14 (StataCorp; College Station, TX, USA), the primary analyses and interaction models by SAS version 9.4 and the inverse probability weight model was fitted by the ipw package of R version 3.4.3 by two of the authors (SK and JH).

The study is registered with the ISRCTN, number 48837681.

### Role of the funding source

The SCORE secretariat was involved in the trial design. The funder of the study had no role in data collection, data analysis, data interpretation, patient recruitment, or writing of the report. The corresponding author had full access to all the data in the study and had final responsibility for the decision to submit for publication.

## Results

**Figure 1:**
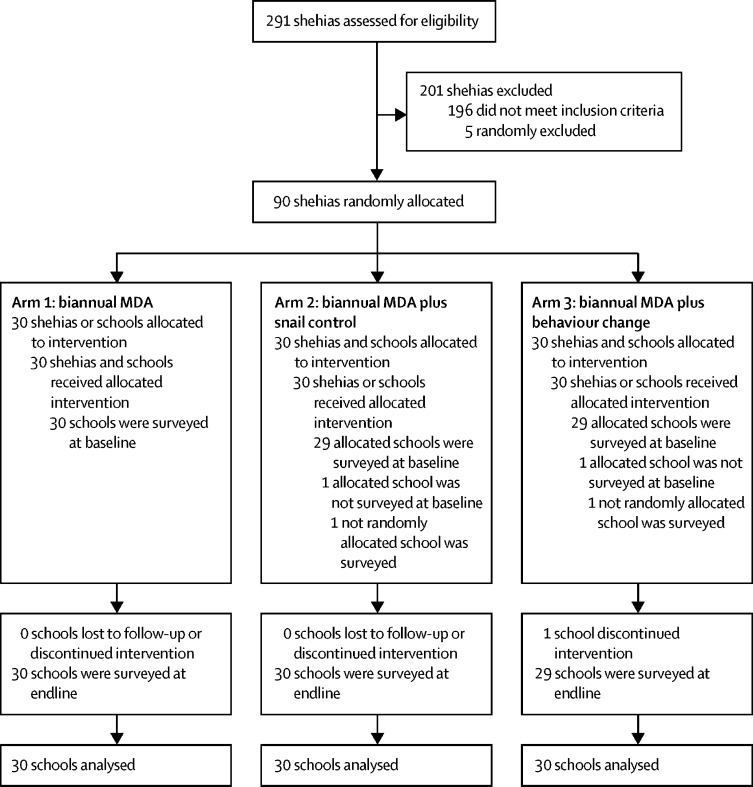
Trial profile Shehia=small administrative region. MDA=mass drug administration.

**Figure 2:**
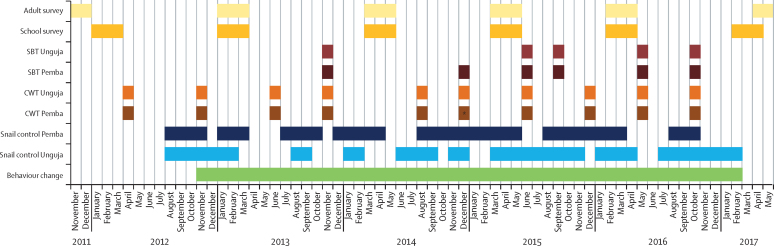
Timeline of interventions and surveys SBT=school-based treatment. CWT=community-wide treatment. *In Pemba, in round 6, community-wide treatment was done by means of health posts.

The study flow and baseline characteristics are indicated in figure 1. The timeline and frequency of all interventions and surveys are illustrated in figure 2. 291 shehias were assessed for eligibility and 45 shehias on each island were randomly allocated to one of three study arms. At baseline, 2853 schoolchildren aged 9–12 years were surveyed from 30 schools in arm 1, 2688 children from 29 schools in arm 2, and 2613 children from 29 schools in arm 3. In arms 2 and 3, a non-randomised school was surveyed, and hence, excluded from further analyses. At the endline survey, 3184 children aged 9–12 years were surveyed from 30 schools in arm 1, 3217 children from 30 schools in arm 2, and 3080 children from 29 schools in arm 3. In arm 3, one school was lost to follow-up since it was transformed into a secondary school. Table 1 indicates between-group differences of the *S haematobium* prevalence in arm 1 (4·2%) and in arm 2 (7·8%), or in arm 3 (6·4%), of the AM egg counts per 10 mL urine in arm 1 (2·8 eggs) and in arm 2 (5·7 eggs), or in arm 3 (5·3 eggs), and of the percentage of heavy infection intensities in arm 1 (0·9%) and in arm 2 (1·8%), or in arm 3 (2·0%). The trial arms were balanced with respect to age and sex of the participants.

**Table 1:** Baseline demographic and clinical characteristics

			**Biannual MDA**	**Biannual MDA plus snail control**	**Biannual MDA plus behaviour change**
Schools*			30	29	29
	Pemba		15	15	15
	Unguja		15	14	14
Total participants			2899	2741	2638
	Pemba		1454 (50·2%)	1308 (47·7%)	1320 (50·0%)
	Unguja		1445 (49·8%)	1433 (52·3%)	1318 (50·0%)
Mean age in years (SD)			10·5 (1·0)	10·5 (1·0)	10·5 (1·0)
	Pemba		10·6 (0·9)	10·7 (1·0)	10·6 (1·0)
	Unguja		10·4 (1·0)	10·4 (1·0)	10·4 (1·0)
Sex					
	Overall				
		Girls	1569	1461	1410
		Boys	1330	1280	1228
	Pemba				
		Girls	822	694	720
		Boys	632	614	600
	Unguja				
		Girls	747	767	690
		Boys	698	666	628
Participants with outcome data			2853	2688	2613
	Pemba		1437 (50·4%)	1276 (47·5%)	1304 (49·9%)
	Unguja		1416 (49·6%)	1412 (52·5%)	1309 (50·1%)
*Schistosoma haematobium* infection†			120/2853 (4·2%)	209/2688 (7·8%)	167/2613 (6·4%)
	Pemba		71/1437 (4·9%)	141/1276 (11·1%)	116/1304 (8·9%)
	Unguja		49/1416 (3·5%)	68/1412 (4·8%)	51/1309 (3·9%)
Arithmetic mean number of eggs per 10 mL of urine			2·8	5·7	5·3
	Pemba		5·0	10·2	9·6
	Unguja		0·6	1·6	1·1
Infection intensity‡					
	Overall				
		Negative	2712/2830 (95·8%)	2451/2658 (92·2%)	2422/2585 (93·7%)
		Light	93/2830 (3·3%)	158/2658 (5·9%)	111/2585 (4·3%)
		Heavy	25/2830 (0·9%)	49/2658 (1·8%)	52/2585 (2·0%)
	Pemba				
		Negative	1363/1433 (95·1%)	1133/1274 (88·9%)	1182/1297 (91·1%)
		Light	47/1433 (3·3%)	102/1274 (8·0%)	70/1297 (5·4%)
		Heavy	23/1433 (1·6%)	39/1274 (3·1%)	45/1297 (3·5%)
	Unguja				
		Negative	1349/1397 (96·5%)	1318/1384 (95·2%)	1240/1288 (96·2%)
		Light	46/1397 (3·3%)	56/1384 (4·0%)	41/1288 (3·2%)
		Heavy	2/1397 (0·1%)	10/1384 (0·7%)	7/1288 (0·5%)

Data are number (%), or n/N (%), unless otherwise stated. MDA=mass drug administration. SD=standard deviation.

* One school in the biannual MDA plus snail control group and one school in the biannual MDA plus behaviour change group were not surveyed at baseline.

† *S haematobium*-positive is defined as urine filtration egg-positive or, in the absence of a urine filtration result, as haematuria-positive (trace, +, ++, and +++).

‡ The intensity of *S haematobium* infection was categorised as negative (0 eggs per 10 mL of urine), light (1–49 eggs per 10 mL of urine), or heavy (≥50 eggs per 10 mL of urine).

**Figure 3:**
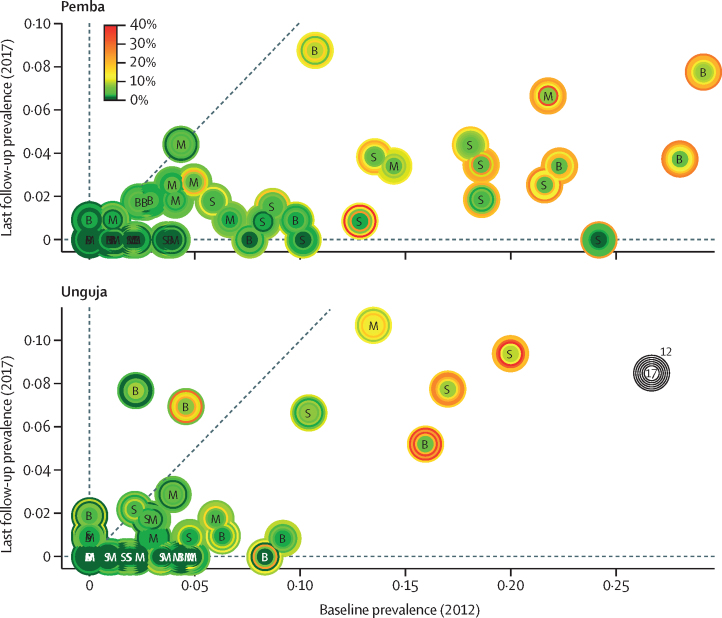
*Schistosoma haematobium* prevalence in 45 schools on each of the two study islands from 2012 to 2017 Colours from red to green indicate the change in prevalence from high to low. Letters indicate the three different study arms. M=biannual praziquantel mass drug administration (MDA) only. B=behaviour change plus biannual praziquantel MDA. S=snail control plus biannual praziquantel MDA.

Table 2 indicates the reduction in prevalence and intensity of infection. The overall *S haematobium* prevalence was reduced from 6·1% in 2012 to 1·7% in 2017, which represents a relative reduction of 72·3%. The percentage of schools with zero infections increased from 17 (19%) of 88 in 2012 to 42 (47%) of 89 in 2017. In 2017, prevalences within schools ranged from 0% to 10·7% (median 0·9%, IQR 0–2·4%). Although most of the 45 schools on each island considerably reduced the prevalence of *S haematobium* from 2012 to 2017, in some years and in some schools prevalences increased compared with the previous year (figure 3). The grand (mean of means) mean of the AM egg counts per 10 mL urine at school level was reduced from 4·7 eggs in 2012 to 1·2 eggs in 2017. The percentage of schools with heavy infection intensities affecting less than 1% of pupils increased from 54 (61%) of 88 in 2012 to 81 (91%) of 89 in 2017.

**Table 2:** Reduction of *Schistosoma haematobium* prevalence and intensity from baseline (2012) to endline (2017)

	**Biannual MDA**	**Biannual MDA plus snail control**	**Biannual MDA plus behaviour change**	**Overall**
Clusters at baseline	30	29	29	88
Tested at baseline with urine filtration and reagent strips*	2853	2688	2613	8154
Tested at baseline with urine filtration*	2830	2658	2585	8073
Tested at baseline with reagent strips*	2852	2681	2613	8146
Infected at baseline*	120	209	167	496
Heavy infection intensity at baseline†	25	49	52	126
Prevalence at baseline*	4·2%	7·8%	6·4%	6·1%
Heavy infection intensity at baseline†	0·9%	1·8%	2·0%	1·6%
Clusters at year 6	30	30	29	89
Tested in year 6 with urine filtration and reagent strips*	3184	3217	3080	9481
Tested in year 6 with urine filtration*	3171	3213	3078	9462
Tested in year 6 with reagent strips*	3183	3198	3078	9459
Infected in year 6*	46	56	58	160
Heavy infection intensity in year 6†	12	8	13	33
Prevalence in year 6*	1·4%	1·7%	1·9%	1·7%
Heavy infection intensity in year 6†	0·4%	0·3%	0·4%	0·4%
Absolute difference between prevalence at year 6 and baseline*	−2·8	−6·0	−4·5	−4·4
Relative difference between prevalence in year 6 and baseline (% change)*	−65·7%	−77·6%	−70·5%	−72·3%
Village level arithmetic mean infection intensity at baseline (including zero egg counts)†	2·8	6·3	5·0	4·7
Village level arithmetic mean infection intensity at year 6 (including zero egg counts)†	1·0	1·0	1·5	1·2
Egg reduction rate (1–year 6 intensity at baseline)†	0·6	0·8	0·7	0·8
Individual-level arithmetic mean infection intensity at baseline (excluding zero egg counts)†	68·0	73·5	84·6	75·9
Individual-level arithmetic mean infection intensity at year 6 (excluding zero egg counts)†	75·4	58·5	78·4	70·6

MDA=mass drug administration.

* *Schistosoma haematobium*-positive is defined as urine filtration egg-positive or, in the absence of a urine filtration result, as haematuria-positive (trace, +, ++, and +++).

† The intensity of *S haematobium* infection was categorised as negative (0 eggs per 10 ml of urine), light (1–49 eggs per 10 mL of urine), or heavy (≥50 eggs per 10 mL of urine).

In 2017, the *S haematobium* prevalence decreased to 1·4% with MDA alone, 1·7% with MDA plus snail control, and 1·9% with MDA plus behaviour change. The generalised estimating equations revealed no significant differences between the prevalence of *S haematobium* with MDA plus snail control (odds ratio [OR] 1·21, 95% CI 0·6–2·7) or MDA plus behaviour change (OR 1·31, 95% CI 0·6–2·9) compared with biannual MDA alone (table 3). Similarly, no significant difference was observed between the infection intensity with MDA plus snail control (OR 0·93, 95% CI 0·3–3·3) or with MDA plus behaviour change (OR 1·44, 95% CI 0·4–4·4) compared with MDA alone. Adjusting for age, sex, and cluster weights did not change the point or interval estimates noteworthily (table 3). Intra-class correlation was estimated at 0·35.

**Table 3:** (Un)adjusted ORs of prevalence and count ratios for infection intensity

			**OR/CR**	**95% CI**	**p value**
**Primary analysis**					
Prevalence 2017					
	Unadjusted (n=9481)				
		MDA	1·0 (ref)	..	..
		MDA + snail control	1·21	0·6–2·7	0·64
		MDA + behaviour change	1·31	0·6–2·9	0·50
	Adjusted (n=9481)*				
		MDA	1·0 (ref)	..	..
		MDA + snail control	1·19	0·5–2·6	0·66
		MDA + behaviour change	1·44	0·7–3·1	0·38
Egg counts 2017					
	Unadjusted (n=9462)				
		MDA	1·0 (ref)	..	..
		MDA + snail control	0·93	0·3–3·3	0·91
		MDA + behaviour change	1·35	0·4–4·4	0·62
	Adjusted (n=9462)*				
		MDA	1·0 (ref)	..	..
		MDA + snail control	0·96	0·3–3·3	0·94
		MDA + behaviour change	1·80	0·6–6·0	0·32
**Exploratory analysis**					
Prevalence 2012 *vs* 2017					
	Intervention × year (n=17 635)†				
		MDA	1·0 (ref)	..	..
		MDA + snail control	0·63	0·4–1·1	0·13
		MDA + behaviour change	0·84	0·5–1·6	0·60
Prevalence 2017					
	Adjusted baseline prevalence (n=9250)				
		MDA	1·0 (ref)	..	..
		MDA + snail control	0·64	0·3–1·3	0·17
		MDA + behaviour change	0·82	0·4–1·9	0·65
	Inverse probability weights (n=9250)‡				
		MDA	1·0 (ref)	..	..
		MDA + snail control	0·65	0·3–1·5	0·31
		MDA + behaviour change	1·06	0·5–2·5	0·95

OR=odds ratio. CR=count ratios.

* Sex and age are also included in the model, along with weighting for number of children who provided data, because not all schools were able to sample 100 children aged 9–12-years.

† Baseline year (2012) is the reference.

‡ Clusters are inversely weighted by their baseline prevalence as continuous variables.

The results of exploratory analysis by means of different models to adjust for imbalance in *S haematobium* prevalence at baseline suggested a greater effect of snail control compared with MDA alone with consistent OR estimates ranging from 0·63 to 0·65. However, 95% CIs were broad and the difference not significant (table 3). Likewise, behaviour change intervention showed slight improvements but the point estimates were less consistent and closer to unity (OR: 0·82–1·06).

In MDA rounds 1, 2, 3, and 5 on both islands and in Unguja also in round 6, children were targeted by CWT. Table 4 shows that the coverage in these rounds, stratified by study arm, ranged from 63·8% to 86·6% as determined by NTD Programme staff. In rounds 4, 7, 8, 9, and 10 on both islands and in Pemba also in round 6, children received praziquantel by SBT. The coverage of SBT ranged from 72·2% to 90·4% when assessed by NTD Programme staff and from 69·8% to 98·1% in the trial coverage surveys.

**Table 4:** Praziquantel treatment coverage in 90 study schools and shehias

		Shehias with schools*	School- children registered in school*	School-children treated*	School- children treated*(%)	**School- children surveyed**†	**School- children treated**†	**School- children treated**†**(%)**	Shehias*	Total population*	Total population treated*	Total population eligible for treatment*	Total population treated*(%)
**2012**													
MDA round 1													
	MDA	..	..	..	..	..	..	..	31	116 746	99 187	..	85·0%
	MDA + snail control	..	..	..	..	..	..	..	31	118 596	97 744	..	82·4%
	MDA + behaviour change	..	..	..	..	..	..	..	30	137 953	105 450	..	76·4%
MDA round 2													
	MDA	..	..	..	..	..	..	..	31	147 511	122 100	..	82·8%
	MDA + snail control	..	..	..	..	..	..	..	31	127 429	104 634	..	82·1%
	MDA + behaviour change	..	..	..	..	..	..	..	30	136 698	118 409	..	86·6%
**2013**													
MDA round 3													
	MDA	..	..	..	..	..	..	..	30	121 089	88 261	99 511	72·9%
	MDA + snail control	..	..	..	..	..	..	..	31	119 681	91 732	104 918	76·6%
	MDA + behaviour change	..	..	..	..	..	..	..	30	118 809	87 221	101 752	73·4%
MDA round 4													
	MDA	25	18 022	13 011	72·2%	3221	2368	73·5%	31	151 775	79 993	95 440	52·7%
	MDA + snail control	23	13 007	9748	74·9%	3262	2276	69·8%	31	128 311	88 621	112 615	69·1%
	MDA + behaviour change	24	25 289	19 220	76·0%	3164	2535	80·1%	30	127 390	88 512	108 168	69·5%
**2014**													
MDA round 5													
	MDA	..	..	..	..	..	..	..	31	128 381	84 837	100 029	66·1%
	MDA + snail control	..	..	..	..	..	..	..	31	135 072	96 332	115 553	71·3%
	MDA + behaviour change	..	..	..	..	..	..	..	30	139 888	91 931	116 218	65·7%
MDA round 6													
	MDA	15	13 023	11 155	85·7%	3276	2661	81·2%	15	61 932	40 419	47 644	65·3%
	MDA + snail control	14	9775	7457	76·3%	3365	2702	80·3%	15	103 447	65 950	80 318	63·8%
	MDA + behaviour change	15	17 433	14 058	80·6%	3230	2709	83·9%	15	66 864	45 481	56 290	68·0%
**2015**													
MDA round 7													
	MDA	31	37 374	31 454	84·2%	..	..	..	31	123 317	68 236	88 501	55·3%
	MDA + snail control	31	40 969	30 999	75·7%	..	..	..	31	138 000	83 869	109 957	60·8%
	MDA + behaviour change	29	40 653	32 045	78·8%	..	..	..	30	129 842	75 649	98 539	58·3%
MDA round 8													
	MDA	31	49 360	36 810	74·6%	3298	3052	92·5%	31	106 996	73 832	93 540	69·0%
	MDA + snail control	31	43 405	32 948	75·9%	3398	3172	93·3%	31	126 034	89 604	114 197	71·1%
	MDA + behaviour change	30	47 702	35 818	75·1%	3114	2872	92·2%	30	114 038	79 227	101 873	69·5%
**2016**													
MDA round 9													
	MDA	31	40 233	35 033	87·1%	..	..	..	31	120 178	48 779	84 304	40·6%
	MDA + snail control	31	42 653	35 995	84·4%	..	..	..	31	128 345	45 045	90 304	35·1%
	MDA + behaviour change	30	50 930	42 862	84·2%	..	..	..	30	115 166	49 222	83 809	42·7%
MDA round 10													
	MDA	30	42 545	38 430	90·3%	3192	3126	97·9%	31	121 190	74 976	90 234	61·9%
	MDA + snail control	30	45 660	39 098	85·6%	3236	3158	97·6%	30	138 817	892 77	107 045	64·3%
	MDA + behaviour change	30	51 386	46 469	90·4%	3093	3034	98·1%	30	134 410	79 978	95 689	59·5%

MDA=mass drug administration.

* Ministry of Health data.

† Cluster-randomised trial data. Coverage in school-based treatment and community-wide treatment in ten rounds of mass drug administration done from 2012 to 2017 was assessed by the Zanzibar Ministry of Health and within our cluster-randomised trial. Calculation of coverage is described in detail in Knopp et al 2016.^18^

In the 15 shehias in Pemba, snail control was applied in a large and constant number of HWCSs identified and visited from 2012 until 2016 (table 5). Annual niclosamide coverage in HWCSs with *Bulinus* spp ranged from 84·0% to 97·6%. In Unguja, additional HWCSs were identified every year. Coverage of infested HWCSs ranged from 31·1% to 86·4%.

**Table 5:** Snail control coverage in 15 intervention shehias on each of the two study islands

	**2012**	**2013**	**2014**	**2015**	**2016**
**Unguja**					
Human water contact sites	39	40	91	105	111
Human water contact sites with *Bulinus* spp	29 (74·4%)	22 (55·0%)	47 (51·7%)	35 (33·3%)	50 (45·1%)
Treated human water contact sites with niclosamide when *Bulinus* spp was found	9 (31·1%)	19 (86·4%)	33 (70·2%)	29 (82·9%)	36 (72·0%)
Total *Bulinus* spp collected	1716	565	676	221	785
Total *Bulinus* spp shedding	0	13	17	0	5
**Pemba**					
Human water contact sites	140	139	143	143	139
Human water contact sites with *Bulinus* spp	45 (32·1%)	46 (33·1%)	45 (31·5%)	42 (29·4%)	29 (20·9%)
Treated human water contact sites with niclosamide when *Bulinus* spp was found	38 (84·0%)	43 (93·5%)	42 (93·3%)	41 (97·6%)	26 (89·7%)
Total *Bulinus* spp collected	2599	788	795	1012	384
Total *Bulinus* spp shedding	4	4	1	0	0

The school-based and classroom-based interventions for behaviour change reached annually several thousand children registered in schools or madrassas in Pemba and Unguja (table 6). The washing platforms installed in 2014 and 2015 were used frequently by all sexes and agegroups. The urinals were not frequently used, probably because of lack of maintenance by the community, and rapidly fell into disrepair.

**Table 6:** Behaviour change activities in 15 intervention schools and shehias on each of the two study islands

		**2012**	**2013**	**2014**	**2015**	**2016**	**2017**
**Total numbers for Pemba**							
Students registered in 15 public primary schools		16 846	NA	NA	NA	NA	17 152
School-based KDEs		15	15	15	15	15	0
Students attending KDE 1–5							
	KDE 1 (2012)	14 364	..	..	..	..	..
	KDE 2 (2013)	..	14 120	..	..	..	..
	KDE 3 (2014)	..	..	15 232	..	..	..
	KDE 4 (2015)	..	..	..	14 923	..	..
	KDE 5 (2016)	..	..	..	..	16 843	..
Classroom-based, interactive teaching		No	Yes	Yes	Yes	Yes	Yes
Community-level behaviour change education meetings		37	30	49	42	49	0
People attending meetings		3160	3289	5581	7071	4191	..
Urinals constructed		0	30	0	0	0	0
Urinals being used		NA	NA	NA	NA	NA	5
Washing platforms constructed		0	0	6	15	0	..
Washing platforms being used		..	..	6	21	20	18*
Madrassa schools involved in intervention		..	..	15	15	54	..
Madrassa teachers trained in intervention		..	..	53	56	82	..
Madrassa students registered in exposed Madrassas		..	..	3591	2066	5735	..
Madrassa KDEs		..	..	15	10	54	..
Madrassa students attending KDE 1–3							
	KDE 1 (2014)	..	..	3129	..	..	..
	KDE 2 (2015)	..	..	..	923	..	..
	KDE 3 (2016)	..	..	..	..	4191	..
**Total numbers for Unguja**							
Students registered in 15 public primary schools		14 887	NA	NA	NA	NA	12 314†
School-based KDEs		6	16	15	12	13	..
Students attending KDE 1–7							
	KDE 1 (2012)	5995	..	..	..	..	..
	KDE 2 (2013)	..	6358	..	..	..	..
	KDE 3 (2013)	..	6248	..	..	..	..
	KDE 4 (2014)	..	..	13 309	..	..	..
	KDE 5 (2014)	..	..	12 625	..	..	..
	KDE 6 (2015)	..	..	..	9886	..	..
	KDE 7 (2016)	..	..	..	..	9577	..
Classroom-based, interactive teaching		No	Yes	Yes	Yes	Yes	Yes
Community-level behaviour change education meetings		0	42	41	60	26	..
People attended meetings		..	988	714	3267	2580	..
Urinals constructed		0	28	0	0	0	..
Urinals being used		..	NA	NA	NA	3	..
Washing platforms constructed		0	0	3	22	0	..
Washing platforms being used		..	NA	NA	NA	19‡	..
Madrassa schools involved in intervention		0	0	0	15	55	..
Madrassa teachers trained in intervention		0	0	0	100	226	..
Madrassa students registered in exposed Madrassas		..	..	..	4507	8647	..
Madrassa KDEs		0	0	0	22	53	..
Madrassa students attending KDE 1–2							
	KDE 1 (2015)	..	..	..	4217	..	..
	KDE 2 (2016)	..	..	..	..	4106	..

NA=not assessed. KDE=Kichocho Day Event.

* Two washing platforms had no water and one needed minor repair.

† One public primary school closed before the end of the study and changed the school type to secondary school only.

‡ The six platforms were not used because the safe water source nearby was no longer functioning (wells dried; tap water has been cut).

## Discussion

Over the past decades, examples from several countries and areas have shown that elimination of schistosomiasis is feasible. Countries having achieved interruption of transmission reported economic improvements, the integrated use of MDA, intermediate host snail control, or improved access to WASH.^5^ We assessed the effect of snail control and behaviour change interventions on top of biannual praziquantel MDA for the reduction of *S haematobium* prevalence and infection intensity among 9–12-year-old children from Zanzibar, one of the first settings in sub-Saharan Africa where interruption of transmission seems to be a feasible goal, in a 5-year repeated cross-sectional cluster-randomised open-label trial. Three key messages emerged from our results. First, biannual MDA alone or in combination with snail control or behaviour change interventions substantially reduced the overall *S haematobium* prevalence and infection intensities and eliminated schistosomiasis as a public health problem from most areas in Zanzibar. Second, biannual MDA was not sufficient to interrupt transmission in 5 years, even if accompanied by additional measures at small scale. Third, there was considerable spatial and temporal heterogeneity of infections.

Of note, although snail control or behaviour change activities did not significantly boost the effect of biannual MDA over the time of the project and at the scale used, they might contribute to further reducing prevalence and enhance interruption of transmission when tailored to focal endemicity, implemented with high coverage and good access to WASH, and applied for a longer period.

The following main challenges should be considered. Although MDA coverage was high in schools, it was low in the community. Non-covered or non-complying individuals might have served as a reservoir of infection contributing to continued transmission. Cure rate (73·6%) and egg reduction rate (94·7%) of praziquantel against *S haematobium* are not perfect.^23^ People are mobile and might have acquired infection in a neighbouring shehia without snail control interventions. Focal application and sporadic coverage of HWCSs with niclosamide to minimise environmental effect does not prevent snails from repopulating the treated freshwater bodies quickly, maintaining the possibility of parasite transmission. Behaviour change needs time to initiate and adopt, and requires access to child-friendly WASH.

Although not as obvious as persistent hotspots in other studies,^24^, ^25^ some pockets with high risk of transmission remained on both islands. These were characterised by a large number of HWCSs containing intermediate host snails and being located in close proximity to schools or settlements.^19^, ^26^ In such high-risk ecological settings, MDA alone might suppress transmission only partially.^27^ Continuing towards the end game of elimination, these areas will need targeted integrated interventions applied with high coverage. To prevent a re-emergence of infection in low-risk and zero-prevalence areas, new tools and strategies tailored to the changing endemic landscape that detect cases and transmission spots with a high sensitivity and trigger interventions that are accepted by a mostly non-infected community are needed. Moving from schistosomiasis control towards elimination as a public health problem and interruption of transmission will require an adaptive strategy, progressing from widespread MDA towards selective interventions and surveillance-response mechanisms.^6^, ^9^, ^28^, ^29^ Translational research to assess the feasibility of combined interventions in hotspot and adequate surveillance-response approaches in low-endemicity areas might provide evidence on how to sustain and further advance the gains made to date, with the ultimate goal of reaching interruption of transmission.

Limitations of our study are that our intervention units were randomly allocated before and not after assessment of the baseline prevalence. Given the low prevalence at the endline survey, our trial was not powered to detect small but biologically important effects as significant differences. Owing to the very low number of positive individuals in this elimination setting, a sufficiently large cluster and participant number was operationally not feasible.^17^ Urine filtration and reagent strip methods are not highly sensitive, particularly if infection intensities are very light.^30^ Use of more rigorous diagnostic approaches and tests with higher sensitivity would probably have resulted in a higher *S haematobium* prevalence and a clearer picture of the real effect of interventions.^31^ Moreover, all of the interventions were implemented and intensified over time and readily available only in 2015. Since we did not assess the abundance and infection of intermediate host snails in shehias outside the snail control arm, it was not possible to compare the number of infected snails across the different arms. As streams and water bodies might run and extend through different shehias, a future control strategy for the whole island should consider treating HWCSs along the whole course of the water body irrespective of the shehia boundaries. Self-reported behaviour change was qualitatively assessed in children by visiting schools in arms 1, 2, and 3 through a mixed methods study at the end of the project (manuscript in preparation). Children targeted by behavioural interventions reported now taking praziquantel during MDA, and having stopped bathing and washing in the river more frequently than children from the other arms (manuscript in preparation). Hence, although no significant difference of added snail control or behavioural change interventions compared with MDA alone was detected in the extremely low *S haematobium* prevalences in our endline survey, the effect of these interventions might be reflected elsewhere.

Urogenital schistosomiasis was eliminated as a public health problem from Zanzibar in more than 90% of the shehias included in the study, but transmission is not yet interrupted and reinfection occurs. It is now necessary to build on the experience gained in the trial, to focus on reducing prevalence in the remaining hotspot areas by biannual MDA plus additional measures implemented with high coverage, and at the scale needed, and to introduce new surveillance-response approaches so that the important gains can be maintained and advanced.

For more on the **R Project** see http://www.r-project.org

## Data sharing

Data collected for the study, anonymised participant data, and a data dictionary defining each field in the set, will be made available to others on reasonable request. De-identified participant data of the requested dataset plus a data dictionary will be made available on reasonable request. The following additional, related documents are published or will be available on reasonable request: published study protocol, statistical analysis plan, informed consent form. These data will be available with publication. The SCORE Data Request Form can be requested from the corresponding author. Data will be shared once the SCORE Data Request Form has been evaluated and signed by all relevant parties.
