# Supplementary material for: Evaluation of integrated interventions layered on mass drug administration for urogenital schistosomiasis elimination: a cluster-randomised trial
Source: Lancet Glob Health. 2019 Jun 26;7(8):e1118–29. doi: 10.1016/S2214-109X(19)30189-5 (PMC6624424; doi:10.1016/S2214-109X(19)30189-5)
Supplement: Supplementary appendix [file mmc1.pdf]

THE LANCET
Global Health

## Supplementary appendix

This appendix formed part of the original submission and has been peer reviewed. We post it as supplied by the authors.

Supplement to: Knopp S, Person B, Ame SM, et al. Evaluation of integrated interventions layered on mass drug administration for urogenital schistosomiasis elimination: a cluster-randomised trial. *Lancet Glob Health* 2019; published online June 26. http://dx.doi.org/10.1016/S2214-109X(19)30189-5.

# Statistical Analysis Plan (SAP) for the Study and Implementation of Schistosomiasis Elimination in Zanzibar (Unguja and Pemba Islands) Using an Integrated Multidisciplinary Approach

**Version 3.1 04 April 2017**

**Authors: Charles King, Jan Hattendorf, Steffi Knopp, David Rollinson and the SAP-Zanzibar Working Group[1]**

## PREFACE

This statistical analysis plan (SAP) has been developed by the Schistosomiasis Consortium for Operational Research and Evaluation (SCORE) team members. It will harmonize the evaluation of outcomes data from the SCORE/Zanzibar Elimination of Schistosomiasis Transmission (ZEST) study of approaches to achieving urogenital schistosomiasis elimination in Zanzibar with the SAP being used in SCORE's studies of gaining and sustaining control of schistosomiasis. It provides guidance on appropriate data sources, variable definitions, and approaches for assessing quantitative outcomes. The intent is to provide consistent approaches to analysis of implementation and outcomes data for each arm of the Zanzibar study, and for the study overall.

The data analysis plan presented below is based on statistical methods that are available using SAS. Use of other statistical analysis packages is also appropriate.

The contents of this SAP represent a minimum analysis for this study. The tables described herein should be completed and provided to SCORE before the conclusion of the study for SCORE to use in reporting to the Bill & Melinda Gates Foundation. They should be available for sharing upon appropriate request, e.g., as supplemental tables for publications. The choice of data to present in published papers is left to authors and will reflect country-specific needs and interests; however, the definitions and approaches described in this document should be used in analysis, and published results should be consistent with those from the SAP analyses. In addition, investigators should review the guidelines from CONSORT (Campbell 2012) to ensure that they address all requirements for reporting randomized trials. Additional exploratory analyses beyond those described here are encouraged.

## Table of Contents

PREFACE ........ 1
I. Study Abbreviations and Brief Definitions ........ 2
II. GOALS AND OBJECTIVES OF THE ZANZIBAR STUDY ........ 3
a. Overview of the Zanzibar study ........ 3
b. Eligibility and randomization ........ 4

[1] Sue Binder, Ye Shen, Christopher Whalen

c. Primary outcomes of interest.......5
d. Study design.......5
III. DATA COLLECTION, AND TYPES OF DATA AVAILABLE.......6
a. Individual-level data.......6
b. Village-level data.......7
IV. SCORE UNIFORM DATASETS.......7
V. DEFINITIONS OF PREVALENCE, INTENSITY, AND COVERAGE.......8
VI. STUDY QUESTIONS AND END-POINTS OF INTEREST FOR THE ZANZIBAR STUDY.......9
a. Key research questions.......9
b. Analyses, tables, and figures to be reported to SCORE by the Approaching Elimination Study researchers.......9
Table 1. Baseline characteristics of participants, by arm.......9
Figure 1. Map of study villages, by arm.......9
Table 2. Coverage and harms.......9
Figure 2. Study flow by arm.......10
Table 3. Descriptive results for baseline and final testing.......10
Figure 3. Intensity categories, by Arm, by Year.......11
Table 4. Comparisons of prevalence and intensity at Year 5 among arms.......11
Figure 4. Village-level mean intensity by year, by arm.......12
VII. PROTOCOL DEVIATIONS AND OTHER ISSUES.......13
REFERENCES.......14
APPENDICES.......14
A. Protocol for the SCORE-ZEST Study.......14
B. Data Elements Available for Analysis.......14
C. Protocol Deviations.......14
D. Data Dictionary.......14
E. Example of a Study Flow Diagram.......14

# I. STUDY ABBREVIATIONS AND BRIEF DEFINITIONS

CDD – Community drug distributor
CWT – Community-wide treatment
GEE - Generalized estimating equations

ICC – Intra-class correlation
MDA – Mass drug administration delivered on a bi-annual basis in Zanzibar at a target dose of 40 mg/kg of praziquantel, with dose estimated from subject height using a dosing pole
MoH – Ministry of Health
NHM – Natural History Museum in London
SAC – school-age children
SBT – School-based treatment
Sh – *Schistosoma haematobium*
SCORE — Schistosomiasis Consortium for Operational Research and Evaluation
SUDS – SCORE Uniform Data Set
Swiss TPH – Swiss Tropical and Public Health Institute
WHO – World Health Organization
Y – Year: Y1 data collection is defined as the baseline data collection, which occurs before treatment. Y1 treatment refers to the first round of MDA treatment
ZAMREC - Zanzibar Medical Research and Ethics Committee
ZEST- Zanzibar Elimination of Schistosomiasis Transmission

# II. GOALS AND OBJECTIVES OF THE ZANZIBAR ELIMINATION STUDY

The overall goal of this project is to provide an evidence base and tools for programmatic decisions on how best to extend control of *Schistosoma haematobium* (Sh) infections toward the goal of local elimination. The SCORE-ZEST study on Zanzibar is a randomized trial designed to inform approaches to mass drug administration (MDA), snail control, and behavioral interventions.

This study involves both Unguja and Pemba islands, the main populated islands of the Zanzibar archipelago belonging to the United Republic of Tanzania. The study design has similarities to that used in SCORE studies focused on gaining and sustaining control of schistosomiasis (Ezeamama, et al., 2016). This Standard Analysis Plan (SAP) draws heavily on the SAP developed for those studies.

The original protocol for the Zanzibar study appears in Appendix A. The list of amendments to the original protocol submitted and approved by the Zanzibar Medical Research and Ethics Committee (ZAMREC) in the years 2012-2017 are presented in Appendix A(b). Appendix B is a table describing the data elements available for analysis from this study.

## a. Overview of the Zanzibar study

At inception, the goals of this project were to:

1. Eliminate schistosomiasis as a public health problem on Unguja in three years and interrupt transmission in five years.
2. Control schistosomiasis throughout Pemba (prevalence <10%) in three years and eliminate it as a public health problem in five years.
3. Identify effective behaviour change strategies with an understanding of the associated costs, motivators, triggers, and barriers associated with behaviour change interventions.

4. Identify effective snail control strategies with an understanding of the associated costs, motivators, triggers, and barriers associated with snail control interventions.

The project involved a randomized control trial to be layered on the planned MDA to be conducted by the Zanzibar National Programme. This trial had 3 study arms:

1. Treatment per the National Plan (twice yearly MDA, including social mobilization and education),
2. Treatment per the National Plan plus snail control, and
3. Treatment per the National Plan plus intensive behavior change interventions.

## b. Eligibility and randomization

A shehia is an administrative subdivision of a district. According to the 2012 population and housing census, Pemba has 121 and Unguja 210 registered shehias. The study includes 45 shehias per island, hence a total of 90 shehias were to be enrolled in the study: 15 shehias per study arm per island. The unit of intervention is the shehia, but the main outcomes were evaluated among schoolchildren in these shehias (i.e., the unit for sampling purposes is the school).

The shehias eligible for the present study were selected ahead of the baseline survey by the following procedure: First, in Unguja, all shehias with no endemic schistosomiasis according to local expert advice (n=104) and in Pemba all shehias without a stream (n=13) were excluded. Second, all shehias without a primary/basic school were excluded (Unguja: n=43; Pemba: n=23). Third, if a shehia had more than one primary/basic school, the school with lower pupil numbers was excluded. Fourth, all shehias with schools that hosted less than 200 pupils in 2008 (latest available data) were excluded (Unguja: n=0; Pemba: n=1). Primary and basic schools in Zanzibar include standards 1-7, consisting of children mainly aged between 7 and 13 years. Hence, we anticipated that, in a school hosting at least 200 students in 2008, at least 100 children aged 9-12 years can be identified in the current study. Fifth, one shehia in Unguja and one shehia in Pemba were excluded as the indicated school was not located in the same shehia. This selection process resulted in exactly 45 eligible shehias in Unguja and 50 eligible shehias in Pemba.
The selection and random allocation of 45 shehias both in Unguja and Pemba to one of the three intervention arms occurred in three steps: First, all eligible shehias having participated in the annual 24-school surveys formerly conducted by the *Piga vita Kichocho* programme (Unguja: n=13; Pemba: n=17) were included in a computer-based randomization to one of the three study arms. Second, out of the remaining 33 shehias in Pemba, 28 were randomly selected to be part of the study. Third, the 32 shehias in Unguja and the 28 shehias in Pemba were randomized to one of the three study arms. Although the randomization may have resulted in differences in the prevalence of *S. haematobium* infection or other factors among study arms at baseline, we did not employ stratified matching or restricted randomization after the initial sample collection and examination.

Children were eligible for inclusion if they: attended the selected schools, were 9-12 years old, and provided written parental consent.

## c. Primary outcomes of interest

Once a shehia was enrolled in the study, the **primary outcomes of interest are prevalence and intensity among 9-12 year-old children**. In addition to 9-12 year-old children, SCORE studies included some data collection on first-year students and adults. More information about data collected from these populations is provided in the study protocol Appendix A, Appendix A(b) and in Appendix B. Their results are not considered in this SAP, but may be used for future secondary analyses.

**Limitations to the analyses included in this SAP:**

- Adverse events during MDAs were recorded by those administering the drugs in a manner consistent with World Health Organization (WHO) or country guidelines. These records were not requested as part of the SCORE research. Therefore, the planned SAP analyses do not include a "safety" component.
- Cost-related data collection has been conducted but analyses are part of another "costing study", which is still ongoing. Therefore, the SAP analyses do not include any cost-effectiveness components.

## d. Study design

**Overview:** The ZEST study is a parallel cluster-randomized, open-label operational research trial of biannual praziquantel MDA vs. biannual MDA plus snail control vs. biannual MDA plus behavior change interventions for the reduction of *Schistosoma haematobium* prevalence in low level endemic communities in Zanzibar.

**Study Arms:** The study has three study arms. The original timeline for conducting the study, including the timing of interventions and data collection, is shown below. Please see Appendix C for a list of known deviations from this plan to date.

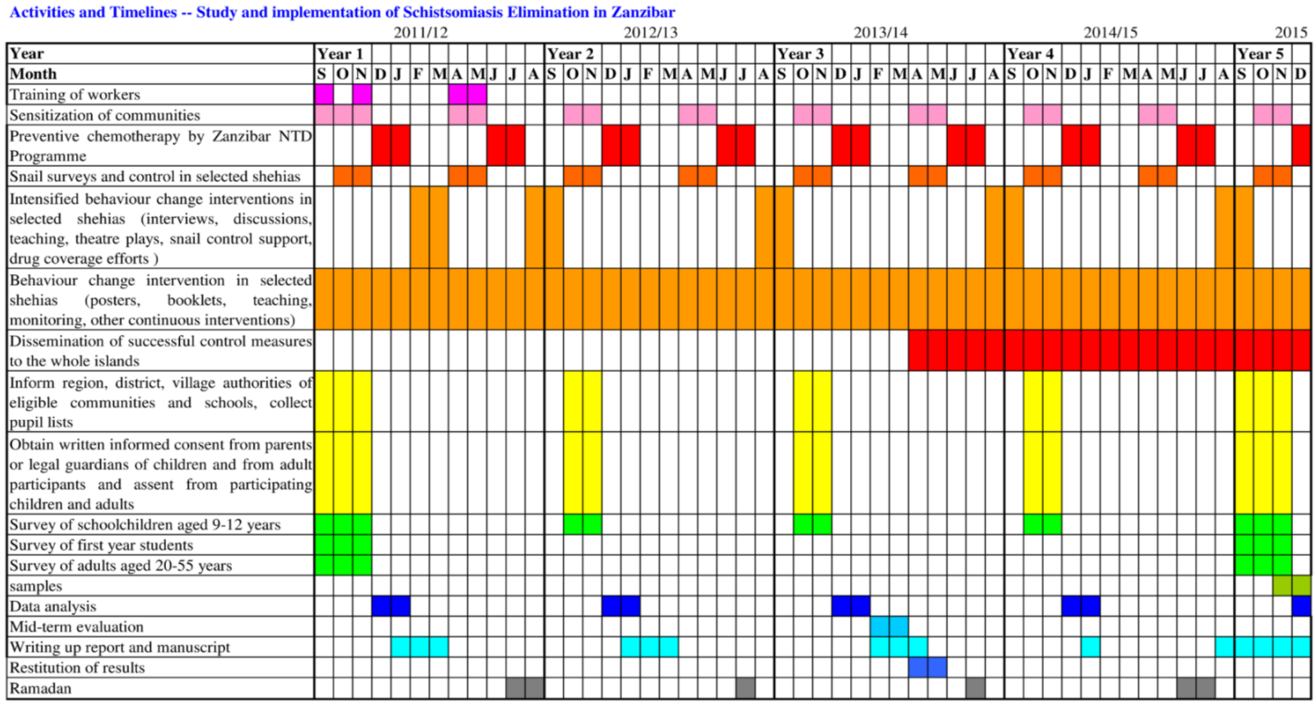


Ramadan 2011: 1 August - 1. September
Ramadan 2012: 21. Juli - 21. August
Ramadan 2013: 9. Juli - 9. August
Ramadan 2014: 28. June - 28. July
Ramadan 2015: 18. June - 18. July

**Populations and expected sample size:** The goal of the study was to enroll 15 eligible shehias per study arm in each of the two main islands of Zanzibar, Pemba and Unguja, and to monitor each shehia's prevalence and intensity of *S. haematobium* infection among **a random sample of 100 children aged 9-12 years old children visiting the main public primary school of each shehia each year**, starting before implementation of bi-annual MDA (Year 1) and continuing through Year 6. Nominal enrollment for the study was 90 shehias, with 9,000 children tested each year.

## III. DATA COLLECTION, AND TYPES OF DATA AVAILABLE

The table in Appendix B describes the data elements available from the Zanzibar study.

### a. Individual-level data

Parasitological data on *S. haematobium* infection among children aged 9-12 are collected annually, starting prior to the first MDA. Data on macro- and microhematuria were collected, but this will not be considered further here because it is not the primary outcome.

In the surveys, 10 ml of urine from a single well-stirred urine sample for each individual are filtered once, and the filters are examined under the microscope; the number of eggs found on each filter is recorded.

Parasitological data were also collected annually on 50 adults per shehia, and at baseline and study end on 100 first-year children per school. However, given that data on these populations do not represent the primary end-points of the SCORE treatment studies, and that the data collected on them are much more limited than that for 9-12 year olds, they will not be reported as primary end-points.

### b. Shehia-level data

Shehia-level data were collected about the area of the shehias (in km$^2$), number of inhabitants, number of primary and secondary schools, number of public and private health centres, number of MCH facilities, water sources for private and domestic use (i.e, piped water, wells, rivers, ponds), sanitation coverage (number of new pit latrines, ventilated improved pit latrines, flush toilets), immigration, emigration and main occupation of the population. Coverage data were collected during each MDA round by the Zanzibar Ministry of Health (MoH). In general, the data on numbers receiving treatment was provided by those administering treatment – either school personnel or community drug distributors (CDDs). However, at some time-points (in 2014, 2015 and 2016), coverage surveys were conducted within the SCORE parasitology survey. The national program administered the MDA on both islands. Denominator data came from a variety of sources, including governmental census reports, shehia leader estimates, and CDDs.

Coverage values for schoolchildren were collected during school-based treatment (SBT) by teachers. Coverage values for the entire population were collected during community wide treatment (CWT) by CDDs. Additionally, we assessed coverage and compliance in schoolchildren and adults included in our annual parasitological SCORE surveys. Coverage is calculated as follows: first, in our SCORE post-MDA survey in the schools and communities, we estimated the proportion of pupils and adults, respectively, who received praziquantel among those who were interviewed and included into our analysis. Second, we calculated the proportion of adults who had received and taken praziquantel among those who were interviewed and included into our analysis. Third, we excluded adults that might have been considered as not eligible for treatment by the MoH (pregnant, breastfeeding or ill) from the analysis and calculated the proportion of individuals who had received praziquantel. Fourth, we again excluded potentially not eligible (pregnant, breastfeeding or ill) adults from the analysis and calculated the proportion of individuals who had received and taken praziquantel. Fifth, with regard to the data obtained from the MoH, we calculated coverage as the proportion of treated individuals among the total population as recorded by the CDDs. Sixth, we calculated coverage as the proportion of treated individuals among the population considered as eligible by the MoH.

Data are also available on snail quantities, species, and schistosomes population genetics from selected shehias in Unguja and Pemba. Measures related to snail control impacts and a range of measures related to the behavior change intervention were also collected.

## *IV. DATASETS*

The data collected in Zanzibar were entered in the country (single entry in Unguja and double entry in Pemba). The data were transferred to the Swiss Tropical and Public Health Institute (Swiss TPH) and the Natural History Museum (NHM) and cleaned by the principal investigator. Issues identified with the data (e.g., missing data, data that appeared to be out of range, etc.) were sent back to the country to be corrected to the extent possible by comparison with original records.

*The following criteria will need to be fulfilled if data are included into the final primary analysis:*

- *Written consent to participate in the study provided by parents*
- *Aged 9-12 years*
- *Complete urine examination (both microhaematuria and urine filtration results are available)*

Each data set is available in SAS, Excel, JMP and .dta formats, and is accompanied by the appropriate data dictionary. The data dictionary also includes metadata pages specific for each study site, indicating issues identified during data collection and cleaning that may need to be taken into account in analysis.

The data dictionary for Zanzibar is in Appendix D.

## V. DEFINITIONS OF PREVALENCE, INTENSITY, AND COVERAGE

The following definitions will be used for individual-level data:

- Individual egg count per 10 ml: Number of eggs found * 10 / volume of urine filtered. If estimated counts are over 1,000 after volume adjustment, they should be truncated at 1,000. Mean eggs per 10 ml will be used for both analysis and reporting, as this measure is both continuous and a standard metric. Note that some software packages may require mean eggs per 10 ml to be rounded prior to analysis, but this will only affect individuals with less than 10ml of urine filtered.
- Egg positive: a child will be deemed to be egg-positive if one or more eggs were found on the filter examined.

The following definitions will be used for reporting on cross-sectional studies:

- **Prevalence of *S. haematobium* infection:** Percentage of egg-positive children among the 9-12 year olds tested in each school each year.
- **Mean intensity of *S. haematobium* infection:** *Arithmetic* mean of individual mean eggs per 10 ml among the 9-12 year-old children. Two values are to be reported:
    - i) <u>School-level intensity</u>: This is the mean egg count for all tested 9-12 year-old subjects (including those with zero egg counts), which is a measure of community-level contamination potential.
    - ii) <u>Individual-level intensity</u>: This is the mean egg count among egg-positive subjects, which is an estimate of the intensity of infection among known active cases.
- **SBT coverage:** Numbers of schoolchildren treated / numbers of schoolchildren.
- **CWT coverage**: Numbers of people in the community treated / number of treatment-eligible people in the community.

# VI. STUDY QUESTIONS AND END-POINTS OF INTEREST FOR THE ZANZIBAR STUDY

The following study questions and endpoints of interest are derived directly from the objectives as stated in the original protocol.

## a. Key research questions

The primary research questions are:

1a. Does the final (Year 6) prevalence of urogenital schistosomiasis among children age 9-12 differ by study arm?

1b. Does the final (Year 6) mean intensity of Sh infection among all children aged 9-12 differ by study arm?

More specifically, the planned analysis will involve a series of arm-to-arm comparisons, prioritized according to what questions are likely to be most important for decision-makers. Since there is risk of “false discovery” of statistically significant association when multiple comparisons are made (Type I error), the number of comparisons formally tested will be restricted. In both studies, the focus of analysis will be on comparison of the current standard of care (twice a year MDA) *versus* twice a year MDA plus snail control *versus* twice a year MDA plus behavior change. P value cutoffs will not be adjusted for multiple comparisons. However, this potential limitation of the analyses will be included in the discussion of results, and interpretation will be guided by the strength of the arm-specific effects.

## b. Analyses, tables, and figures to be reported to SCORE by the ZEST Study researchers

The following describes the tables and figures expected from SCORE studies, as well as approaches to analyzing the data and examples of SAS codes.

### Table 1. Baseline characteristics of participants, by arm.

This would include number of shehias and schools; number of participants; and age, sex, *S. haematobium* prevalence, and intensity assessed both by including all children and including only egg-positive children. Where appropriate, measures of dispersion should be included, for example, interquartile measures of intensity.

### Figure 1. Map of study shehias and schools, by arm.

This can be presented as a single map, with different symbols for each of the arms, or as a series of maps.

### Table 2. Coverage.

| | | **SAC treated** | **SAC total** | **% SAC treated** | **Total population treated** | **Total population eligible for treatment** | **% total eligible population treated** |
|---|---|---|---|---|---|---|---|
| **Year 1** | | | | | | | |
| | **Arm 1** | | | | | | |

| | | | | | | | |
|---|---|---|---|---|---|---|---|
| | **Arm 2** | | | | | | |
| | **Arm 3** | | | | | | |
| **Year 2, etc.** | | | | | | | |
| | **Arm 1** | | | | | | |
| | **Arm 2** | | | | | | |
| | **Arm 3** | | | | | | |
| | **[…]** | | | | | | |

In addition to the table, information should be provided about

- How numerators and denominators were collected, by year if they changed over time
- Any particular issues or concerns related to MDA coverage
- A listing of the serious adverse events or harms

### Figure 2. Study flow by arm.

The study flow diagram describes the process through the phases of a randomized trial. A sample study flow diagram and accompanying table are shown in Appendix E.

### Table 3. Descriptive results for baseline and final testing.

| | **Arm 1** (bi-annual MDA only) | **Arm 2** (bi-annual MDA plus snail control) | **Arm 3** (bi-annual MDA plus behavior change) |
|---|---|---|---|
| **No. tested at baseline** | | | |
| **No. infected at baseline** | | | |
| **Prevalence at baseline** | | | |
| **No. tested at Year 6** | | | |
| **No. infected at Year 6** | | | |
| **Prevalence at Year 6** | | | |
| **Absolute difference between prevalence at Year 6 and baseline** | | | |
| **Relative difference between prevalence at Year 6 and baseline (% change)** | | | |
| **Shehia/school-level arithmetic mean infection intensity at baseline** | | | |
| **Shehia/school level arithmetic mean infection intensity at Year 6** | | | |
| **Egg reduction rate (1-Year 6 intensity/baseline)** | | | |
| **Individual-level arithmetic mean** | | | |

| | | | |
|---|---|---|---|
| **infection intensity at baseline** | | | |
| **Individual-level arithmetic mean infection intensity at Year 6** | | | |

### Figure 3. Intensity categories, by Arm, by Year.

This is a stacked bar chart, showing low, medium, and high intensity infections, by Arm, for Years 1-6. Intensity categories are defined as:

- Sh: Low 1-49 eggs/10 ml, high $\geq$ 50 eggs/10 ml

A supplemental table including the numbers used to make the chart should be available, in case modelers or other analysts want to use the exact data.

### Table 4. Comparisons of prevalence and intensity at Year 6 among arms.

This table describes the primary outcomes.

| | **Unadjusted prevalence model estimate (CI)** | **Adjusted prevalence model estimate (CI)** | **Unadjusted intensity ratio (CI)** | **Adjusted intensity ratio (CI)** |
|---|---|---|---|---|
| **Arm 1 v. Arm 2** | | | | |
| **Arm 1 v. Arm 3** | | | | |
| **Arm 2 v. Arm 3** | | | | |

*General approach to analysis:* The general approach is to use Generalized estimating equations (GEEs) to estimate differences between the arms in year 6 only. We will report unadjusted estimates – with just school and arm fitted in the model – and adjusted estimates – where sex and age are also included in the model, along with weighting for number of children who provided data, because not all shehias/schools were able to sample 100 9-12 year old children.

Intra-class correlation (ICC) will be calculated using mixed models consistent with the GEE setup in the primary analysis.

All models will be based on individual level data on 9-12 year old children only.

*Sample code:* The following codes are provided as examples. Different studies may require further modification of the code below or may require different approaches.

*Unadjusted prevalence*

The evaluation of unadjusted prevalence uses a binomial GEE with logit link function. `school` is treated as the repeated subject, and we assume compound symmetry within a village. 'estimate' is used to test the pre-specified differences between arms.

```
ods trace off;
proc genmod data=sth descending;
    where Year = 2017;
    class intervention school/ param=glm;
    model shpos = intervention / dist=bin link=logit;
    repeated subject=school / type=ind;
    lsmeans intervention  / cl;
    estimate "Snail+MDA vs. MDA" intervention  0 -1  1 / exp;
    estimate "Behave+MDA vs. MDA" intervention  1 -1  0 / exp;
run;
```

*Adjusted prevalence*

*The code is the same as for unadjusted prevalence, but with age, sex, and a weighting for village size added to the model.*

*villweight of individual = 1/# samples collected in village*

```
ods trace off;
proc genmod data=sth descending;
      where Year = 2017;
      class intervention school/ param=glm;
      model shpos = intervention / dist=bin link=logit;
      repeated subject=school / type=ind;
      weight villweight;
      lsmeans Study_Arm / cl;
run;
```

*ICC of prevalence*

```
/*NLMIXED has no where statement prepare data with data from final year
and only the 2 arms for the primary comparison (coded 0/1) */
PROC NLMIXED DATA=sth_yrfin_arm12;
  PARMS b0=0.1 b1=0.1 s2u=0.1;
  xb = b0 + u + interv*b1;
  p = exp(xb)/(1+exp(xb));
  model shpos ~ binary(p);
  random u ~ normal(0,s2u) SUBJECT=school;
  ESTIMATE 'icc' s2u/(3.29 + s2u);
run;
```

*Unadjusted intensity*

*The code is the same as for unadjusted prevalence, but the distribution is changed to negative binomial and link is changed to log.*

*Adjusted intensity*

*The code is the same as for the adjusted prevalence, but the distribution is changed to negative binomial and link is changed to log.*

*Figure 4. Village-level mean intensity by year, by arm.*

*Provide a line graph showing arithmetic mean intensity, by Year, by Arm.*

## VII. PROTOCOL DEVIATIONS AND OTHER ISSUES

A full listing of known protocol deviations is in Appendix C.

Several issues have arisen during the collection of the SCORE datasets. These include:

- Limited enrollment in some school: The target study population size was 100 9-12 year old children. In some few schools, the enrollment was significantly less. To account for this, a “school-weight” term will be added to the GEE model to weight results according to numbers of children tested per school.
- Missing data on some children
  - Children without data on consent, age, sex, and presence or absence of *S. haematobium* eggs are not included in the analyses.
- Irregularities in MDAs
  - SBT was only implemented in MDA Round 4 in November 2013
  - SBT was not conducted in Round 5 in 2014 due to financial constraints on both islands
  - SBT was conducted in Rounds 4, 6, 7, 8, 9 and 10 in Pemba, but not in Rounds 1, 2, 3, and 5
  - SBT was conducted in Rounds 4, 7, 8, 9 and 10 in Unguja, but not in Rounds 1, 2, 3, 5 and 6; this might explain higher prevalences in 2015 compared with 2014
  - SBT in Round 8 was conducted before the elections, in Sept 2015 on both islands; this resulted in a slightly longer re-infection period until the parasitology survey was conducted from February 2016 onwards
  - CWT in Round 6 in Pemba was done with health posts and not door to door, which resulted in a low coverage. This might explain high prevalences in adults in 2015.
  - Pemba started to include madrassa and secondary schools in round 7 in 2015 and from then considerably increased the number of treated children until 2017.
- Confusion on schools and shehias
  - Intv 1 (MDA): Makoba school in Makoba shehia is surveyed in 2012 till 2016, but Makoba was not in list for randomization; hence we exclude it from the primary analysis.
  - Intv 1 (MDA): Kibweni school belongs to Mwanyanya shehia, but was only opened and surveyed only from 2014 onward; hence we exclude it from the primary analysis.
  - Intv 1 (MDA): Bububu shehia was not randomized (but the neighboring Mwanyanya shehia). However, Bububu school was surveyed for Mwanyanya shehia from 2012 onwards till 2017. Hence we keep Bububu school in primary analysis.
  - Intv 1 (MDA): Chaani Mcheza Shauri shehia was not randomized, but adults surveyed annually from 2011 instead of adults from Chaani Kubwa shehia. Chaani Kubwa shehia was randomized to behaviour arm, but adults only surveyed from 2013. No school exists in Chaani Mcheza Shauri, Chaani Kubwa school was randomized and surveyed from baseline and is kept in the primary analysis.
  - Intv 2 (Snail): Kiongwe school blongs to Mafufuni shehia and was randomized but was only surveyed from 2013 onwards. We keep it in the primary analysis.

- Intv 3 (Behaviour): Machui schools belongs to Koani shehia and was surveyed from 2012 onwards; but it was not in the original randomization list and the behaviour team did not work there at the beginning. We exclude it from the primary analysis.
- Intv 3 (Behaviour): Mwera schools belongs to Koani shehia and was surveyed from 2012 onwards; Since it was in the original randomization list, it remains in primary analysis.
- Intv 3 (Behaviour): Regeza Mwendo schools belongs to Mwera shehia and was only surveyed from 2013 onwards. Since it was in the original randomization list, it remains in primary analysis.
- Intv 3 (Behaviour): Mwanakerekwe B school in Mwanakerekwe shehia became a secondary school from 2016; hence it was only surveyed from 2012-2015, but remains in primary analysis.

## REFERENCES

Campbell MK, Piaggio G, Elbourne DR, Altman DG, et al. Consort 2010 statement: extension to cluster randomized trials. BMJ 2012;345:e5661.

Ezeamama AE, He CL, Shen Y, Yin XP, Binder SC, Campbell CH, Jr., et al. Gaining and sustaining schistosomiasis control: study protocol and baseline data prior to different treatment strategies in five African countries. BMC Infect Dis. 2016;16(1):229. doi: 10.1186/s12879-016-1575-2.

## APPENDICES

### A. Protocol for the SCORE-ZEST Study (2011)

### A (b). Amendments of study protocol from 2011 till 2017

### B. Data Elements Available for Analysis

### C. Protocol Deviations

### D. Data Dictionary

### E. Example of a Study Flow Diagram
